# Effect of Microbial Status on Hepatic Odd-Chain Fatty Acids Is Diet-Dependent

**DOI:** 10.3390/nu13051546

**Published:** 2021-05-04

**Authors:** Karolin Weitkunat, Christopher A. Bishop, Maria Wittmüss, Tina Machate, Tina Schifelbein, Matthias B. Schulze, Susanne Klaus

**Affiliations:** 1 Department Physiology of Energy Metabolism, German Institute of Human Nutrition Potsdam-Rehbruecke (DIfE), 14558 Nuthetal, Germany; christopher_allen.bishop@dife.de (C.A.B.); maria.wittmuess@dife.de (M.W.); tina.machate@dife.de (T.M.); klaus@dife.de (S.K.); 2 Research Group Intestinal Microbiology, German Institute of Human Nutrition Potsdam-Rehbruecke (DIfE), 14558 Nuthetal, Germany; tina.schifelbein@dife.de; 3 Department Molecular Epidemiology, German Institute of Human Nutrition Potsdam-Rehbruecke (DIfE), 14558 Nuthetal, Germany; mschulze@dife.de; 4 Institute of Nutritional Science, University of Potsdam, 14469 Potsdam, Germany

**Keywords:** pentadecanoic acid (C15:0), heptadecanoic acid (C17:0), type-2-diabetes, fatty acid synthesis, acetate, propionate, probiotics, gut microbiota, prebiotics, inulin

## Abstract

Odd-chain fatty acids (OCFA) are inversely associated with type-2-diabetes in epidemiological studies. They are considered as a biomarker for dairy intake because fermentation in ruminants yields high amounts of propionate, which is used as the primer for lipogenesis. Recently, we demonstrated endogenous OCFA synthesis from propionate in humans and mice, but how this is affected by microbial colonization is still unexplored. Here, we investigated the effect of increasing microbiota complexity on hepatic lipid metabolism and OCFA levels in different dietary settings. Germ-free (GF), gnotobiotic (SIH, simplified human microbiota) or conventional (CONV) C3H/HeOuJ-mice were fed a CHOW or high-fat diet with inulin (HFI) to induce microbial fermentation. We found that hepatic lipogenesis was increased with increasing microbiota complexity, independently of diet. In contrast, OCFA formation was affected by diet as well as microbiota. On CHOW, hepatic OCFA and intestinal gluconeogenesis decreased with increasing microbiota complexity (GF > SIH > CONV), while cecal propionate showed a negative correlation with hepatic OCFA. On HFI, OCFA levels were highest in SIH and positively correlated with cecal propionate. The propionate content in the CHOW diet was 10 times higher than that of HFI. We conclude that bacterial propionate production affects hepatic OCFA formation, unless this effect is masked by dietary propionate intake.

## 1. Introduction

Pentadecanoic acid (C15:0) and heptadecanoic acid (C17:0) are the main odd-chain fatty acids (OCFA) in human plasma, but account for only a small proportion of long-chain fatty acids in phospholipids. The majority of research focuses on their role as biomarkers for dietary food intake assessment and disease risk. In humans, increased OCFA levels in plasma phospholipids have been linked to decreased fasting blood glucose levels [1,2], improved insulin resistance [3], and a lower incidence of type-2-diabetes mellitus [4,5].

It is well documented that ruminants can synthesize OCFA through bacterial fermentation in the rumen and de novo fatty acid synthesis using propionate-derived propionyl-CoA instead of acetyl-CoA as the primer for fatty acid synthesis [6]. OCFA absorbed from the intestine can be utilized in the mammary gland, resulting in its incorporation into milk fat. Therefore, OCFA have been suggested as potential biomarkers for dairy intake [5,7]. This relies on the assumption that OCFA are not synthesized endogenously by mammals, and therefore solely reflect the intake of dairy products [8,9]. Interestingly, it is well acknowledged that there is no decisive evidence that plasma concentrations of OCFA reflect their dietary consumption. The plasma ratio of C15:0 to C17:0 is approximately 1:2, which is opposite to the expected ratio of approximately 2:1 detected in dairy fat [10]. Therefore, the possibility of endogenous production is being discussed. The work of Jenkins et al. described an endogenous formation of OCFA via α-oxidation. It takes place in peroxisomes where the enzyme 2-hydroxyacyl-CoA lyase 1 (Hacl1) is responsible for the cleavage of one carbon from even-chain fatty acids, resulting in the formation of OCFA [11]. Further, we have previously shown that propionate is a direct precursor of OCFA formation in humans [12] and rodent models [2,13]. Propionate derives mainly from the bacterial fermentation of dietary fibers and is likely a relevant source that increases hepatic and circulating OCFA. However, there are other nutritional components, such as the amino acid valine, which can also contribute to a higher endogenous propionyl-CoA pool through its catabolism [14], but also induces Hacl1 expression and thereby affects OCFA formation [15].

Yet, to the best of our knowledge, whether different intestinal microbial compositions can affect the formation of OCFA in rodent models has been poorly investigated. There is only one study that shows no differences in circulating OCFA levels in the presence or absence of intestinal microbiota in mice on semi-synthetic diets [16]. However, the microbial population and OCFA induction can be influenced by a wide range of variables, such as diet composition, short-chain fatty acid (SCFA) concentrations and hepatic metabolism, and these relationships have not yet been addressed. Therefore, here we investigated if and how hepatic OCFA are affected by differences in diet composition, the intestinal fermentation pattern and bacterial colonization in mice. This can have obvious implications for the interpretation of epidemiological studies. As the fermentation product propionate is likely a relevant source of circulating OCFA, the described positive association of plasma OCFA and a decreased type 2 diabetes risk could also be attributed to changes in microbial composition. As described previously [12], the liver is the main organ of OCFA synthesis and very well reflects circulating concentrations. If hepatic OCFA synthesis is also influenced by other factors, such as the intake of dietary fibers or microbial composition, this would indicate that OCFA levels are a poor marker for the intake of dairy products.

To address this, we first used C3H mice with different microbial colonization on a CHOW diet to investigate the differences in OCFA formation due to bacterial communities related to a standard housing diet. This CHOW diet includes relatively high amounts of polysaccharides, which may contain fermentable substrates. To elucidate whether microbial status might be responsible for changes in OCFA formation, we compared germ-free mice, conventional mice and a gnotobiotic mouse model with a known and less complex bacterial community. As such, it was much easier to analyze specific aspects of host–bacteria interactions under highly reproducible conditions. More precisely, C3H-mice harboring a simplified human intestinal microbiota (SIHUMI) of eight bacterial species (*Anaerostipes caccae, Bacteroides thetaiotaomicron, Bifidobacterium longum, Blautia producta, Clostridium butyricum, Clostridium ramosum, Escherichia coli* and *Lactobacillus plantarum*) were used [17]. In a second experiment, we investigated the effect of a semi-synthetic high-fat (HFI) diet, such as that routinely used for diet-induced obesity [18,19]. For the investigation of dietary fiber-mediated effects, the high-fat diet was supplemented with inulin, a highly fermentable fiber, which induces microbial fermentation leading to an increased production of SCFA (acetate, propionate and butyrate) [18]. As such, we used two commonly used rodent diets that both have a high fiber content but have highly different dietary compositions and metabolic effects, especially on liver metabolism, in order to assess the possible interaction of diet composition and microbial colonization and the effect on hepatic OCFA formation.

## 2. Materials and Methods 

Animal experiment: Male C3H/HeOuJ mice were bred in the Max-Rubner Laboratory. Germ-free mice were colonized with a simplified human intestinal microbiota (SIHUMI) consisting of eight bacterial species (*Anaerostipes caccae, Bacteroides thetaiotaomicron, Bifidobacterium longum, Blautia producta, Clostridium ramosum, Clostridium butyricum, Escherichia coli* and *Lactobacillus plantarum*). The remaining mice were born either germ-free or with conventional colonization. All mice were kept individually in positive-pressure isolators (Metall & Plastik, Radolfzell, Germany) at 22 ± 2 °C and a relative air humidity of 55 ± 5% on a 12 h light/dark cycle. Mice consumed ad libitum a CHOW diet (Altromin fortified 1314, containing 18 energy% fat, 27 energy% protein and 55 energy% carbohydrates, Altromin, Lage, Germany) and autoclaved distilled water. At 12 weeks of age, an additional cohort of mice was switched to the experimental high-fat diet supplemented with 7% inulin (containing 42 energy% fat, 24 energy% protein and 34 energy% carbohydrates, for detailed diet composition see [2]) for 4 weeks of intervention. Animals were euthanized in the morning (8–10 a.m.) with isoflurane and peripheral blood was obtained by cardiac puncture. Tissues were removed, weighed and immediately frozen in liquid nitrogen before they were stored at −80 °C. All experiments were approved by the animal welfare committee of the Ministry of Agriculture and Environment (approval code 2347-15-2017; State of Brandenburg, Germany).

NMR and food intake: Body composition was determined non-invasively with a nuclear magnetic resonance spectrometer, EchoMRI (Echo Medical Systems), after four weeks of intervention. Lean mass was calculated by subtracting body fat mass from body weight. Food intake was measured by a weekly weighing of the diet using a spring balance.

Triglyceride measurement: Triglyceride concentrations were measured in frozen liver tissue. Liver tissue (40 mg) was homogenized in 1 mL of 10 mM sodium phosphate buffer (pH 7.4) containing 1 mM EDTA and 1% polyoxyethylene-10-tridecylethan. Triglyceride (Triglyceride Determination Kit, Merck KGaA, Darmstadt, Germany) and protein (DC Protein Assay; Bio-Rad Laboratories GmbH, Feldkirchen, Germany) concentrations were analyzed according to manufacturers’ instructions in triplicates.

Gene expression analysis: Liver tissue (20 mg) and colon mucosa (20 mg) were ground in liquid nitrogen. RNA extraction (peqGOLD TriFast, VWR International GmbH, Darmstadt, Germany), DNase treatment (Turbo DNA-free Kit; Ambion, Kassel, Germany), cDNA synthesis (RevertAid H Minus First Strand cDNA Synthesis Kit; Fermentas GmbH, St. Leon-Rot, Germany) and qRT-PCR (SYBR Green Universal PCR Master Mix; Applied Biosystems, Thermo Fisher Scientific GmbH, Dreieich, Germany) were performed as previously described [18]. Gene expression analysis was performed by qPCR with the ViiA™ 7 Real-Time PCR System (Applied Biosystems). The sequences of primers are summarized in supplementary Table S1. Gene expression was calculated as ddCT, using Hprt or Actin as the reference gene. Data were normalized to the CONV group, which was set to a value of 1.

Western blot: The isolation, immunoblotting and detection of proteins was performed as previously described [13]. As primary antibodies, SLC22A7 (Proteintech, 26796-1-AP), CD36 (R&D, MAB2519), ACSS2 (Invitrogen, PA5-26612), ACSS3 (Proteintech, 16204-1-AP), FASN (CST, 3189), ELOVL6 (Proteintech, 21160-1-AP), SCD1 (SC, 515844), and HACL1 (Sigma, HPA035496) were used. The following antibodies were used as secondary antibodies: anti-rabbit IgG (CST, 7074), anti-rat IgG (SC, 2032) and anti-mouse IgG (CST, 7076). Protein expression was normalized to GAPDH (Ambion, AM4300) or ponceau staining. Western blot densitometry was quantified using ImageJ software and the data were normalized to CONV group.

Fatty acid analysis: The long-chain fatty acid (LCFA) composition of liver phospholipid fraction was analyzed by extraction with tert-butyl methyl ether/methanol (2/1, *v*/*v*), solid phase separation, hydrolysis and methylation with trimethyl sulfonium hydroxide (TMSH), and subsequent analysis by gas chromatography as already validated and described [18]. Short-chain fatty acid (SCFA; acetate, propionate and butyrate) concentrations were measured in cecum contents using gas chromatography as described [18]. 

Statistical analysis: The data are presented as mean ± SEM. Statistical calculation was performed using GraphPad Prism 8 (GraphPad Software, La Jolla, CA, USA). The Kolmogorov–Smirnov test was used to test the normal distribution and homogeneity of variances. Normally distributed data were analyzed with an ordinary one-way analysis of variance (ANOVA) followed by Bonferroni’s post-test. A nonparametric Kruskal–Wallis test was used to analyze non-normally distributed data. Differences with a *p*-value less than 0.05 were considered statistically significant. Principal component analysis was performed using GraphPad Prism 9 (GraphPad Software, La Jolla, CA, USA). Data were standardized to have a mean of 0 and a standard deviation of 1. Principle components were selected by parallel analysis, which accounts for variances in the data due to random error or noise, and according to the highest eigenvalues (CHOW: PC1, 7.3; PC2, 3,4; PC3, 2.4; HFI: PC1, 5.1; PC2, 3.7; PC3, 2.0)

## 3. Results

### 3.1. Phenotype and Hepatic Lipid Metabolism of Mice Harboring Different Microbial Status

#### 3.1.1. CHOW Fed Mice

20-week-old mice on CHOW showed phenotypical differences according to their microbial colonization (Table 1). Germ-free mice (GF) had a lower body weight than SIHUMI mice (SIH) and conventional mice (CONV), in line with a lower tissue weight of the liver, gastrocnemius muscle, and fat tissues (subcutaneous WAT, epididymal WAT, BAT). As a largely distended cecum due to high liquid content is a known characteristic of germ-free rodents [20], the cecum weight was highest in GF and lowest in CONV (Table 1).

**Figure 1:**
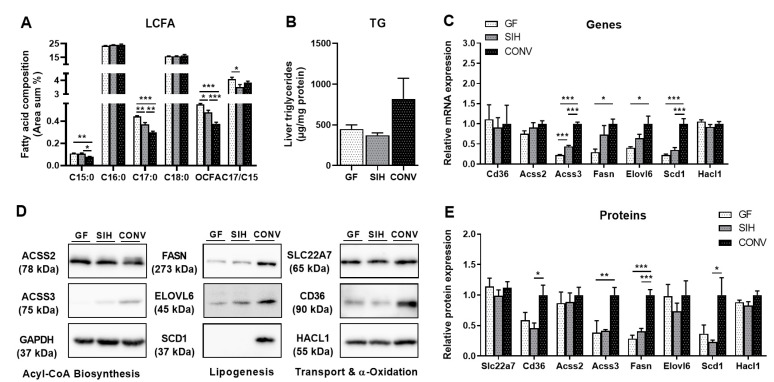
Germ-free mice (GF) and SIHUMI mice (SIH) on CHOW diet display a reduced hepatic de novo fatty acid synthesis, but increased relative OCFA compared to conventional mice (CONV). Twenty-week-old male C3H/HeOuJ mice on CHOW diet (Altromin fortified 1314): (**A**) long-chain fatty acid (LCFA) composition of hepatic phospholipid fraction. Results are expressed as area percentage of individual fatty acids to total area of detected fatty acids. (**B**) Hepatic triglyceride (TG) concentration. Analysis of fatty acid metabolism with both (**C**) mRNA and (**E**) protein expression. (**D**) Representative Western blots for fatty acid signaling. Data are mean + SEM (*n* = 6) and expression data were normalized to CONV group. Hprt was used as the housekeeping gene and GAPDH as the loading control for the protein. * *p* < 0.05; ** *p* < 0.01; *** *p* < 0.001.

Gut microbiota, in concert with the diet, has been reported to have regulatory functions in the hepatic lipid metabolism and lipid levels in tissues. However, the influence of microbial status on OCFA has been poorly examined. Here, the difference in OCFA formation as dependent on microbial status was first examined between GF, SIH and CONV on a CHOW diet. Surprisingly, conventional mice displayed a significantly lower relative formation of pentadecanoic acid (C15:0) and heptadecanoic acid (C17:0). Thus, the proportion of OCFA in liver phospholipids was about 32% and 21% lower in CONV compared to GF and SIH, respectively (Figure 1A). Although the relative formation of OCFA was the lowest in CONV, these mice tended (*p* = 0.15) to have higher hepatic triglyceride levels than the other groups (Figure 1B). This trend was reflected in the significant differences in the hepatic expression profiles of enzymes related to hepatic lipid metabolism. Key enzymes of de novo fatty acid synthesis, such as Acyl-CoA synthetase short chain family member 3 (Acss3), fatty acid synthase (Fasn), elongation of very long-chain fatty acids protein 6 (Elovl6), and stearoyl-CoA desaturase 1 (Scd1), showed the lowest gene expressions in GF, and the highest expressions in CONV (Figure 1C). Protein expression by Western blot analysis confirmed these differences, with the exception of ELOVL6, which showed no significant differences in protein expression between the groups (Figure 1D,E). While the mRNA expression of the fatty acid translocase Cd36 was not altered (Figure 1C), its protein expression was the highest in CONV (Figure 1D,E). Of note, the gene and protein expression of peroxisomal 2-hydroxyacyl-CoA lyase (Hacl1), a key enzyme of α-oxidation, which has been proposed to play a role in endogenous C17:0 biosynthesis [11], was not affected by the microbial status. Similarly, the protein expression of solute carrier family 22 member 7 (Slc22a7), which is a hepatic propionate transporter, was unchanged.

#### 3.1.2. HFI Fed Mice

After the four-week high-fat diet challenge with the highly fermentable dietary fiber inulin (HFI), there were no differences in food intake or final body weight between the groups (Table 2). Body composition, as determined by NMR, showed the highest total fat mass in SIH-colonized mice (without statistical significance), which mice also showed significantly increased epididymal white adipose tissue (eWAT) and subcutaneous white adipose tissue (sWAT) weights compared to GF. In contrast, lean mass was the highest in CONV, as reflected by the higher gastrocnemius muscle and liver tissue weights in this group. Furthermore, the brown adipose tissue (BAT) weight was increased in CONV. Consistent with the data on CHOW-fed mice, the cecal weight was reduced with increasing colonization (Table 2).

**Figure 2:**
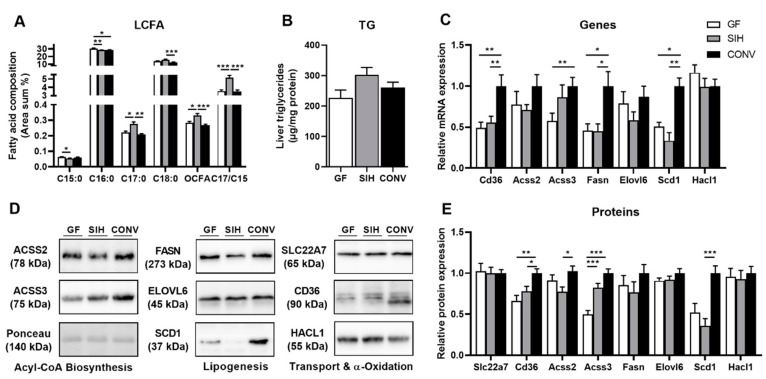
Germ-free mice (GF) and SIHUMI mice (SIH) on high-fat inulin diet (HFI) display reduced hepatic de novo fatty acid synthesis compared to conventional mice (CONV), while total OCFA levels are the highest in SIH mice. Sixteen-week-old male C3H/HeOuJ mice on HFI diet for 4 weeks: (**A**) long-chain fatty acid (LCFA) composition of hepatic phospholipid fraction. Results are expressed as area percentage of individual fatty acids to total area of detected fatty acids. (**B**) Hepatic triglyceride (TG) concentration. Analysis of fatty acid metabolism with both (**C**) mRNA and (**E**) protein expression. (**D**) Representative Western blots for fatty acid signaling. Data are mean + SEM (*n* = 8–10) and expression data were normalized to CONV group. Actin was used as the housekeeping gene and ponceau staining as the loading control for protein. * *p* < 0.05; ** *p* < 0.01; *** *p* < 0.001.

To increase microbial SCFA production, we used a high-fat diet supplemented with inulin (HFI) to investigate the relationship between microbial composition and hepatic lipid metabolism. Surprisingly, SIH colonization resulted in the lowest C15:0 level but the highest C17:0 level in liver phospholipids (25% increase compared to CONV), resulting in the highest total OCFA levels in SIH animals, while there were no differences between GF and CONV (Figure 2A). Further, there were no differences in liver triglycerides (Figure 2B), but the gene and protein expressions of the enzymes involved in hepatic lipid transport and lipogenesis showed a clear increase in CONV mice. The mRNA and protein expressions of fatty acid translocase (Cd36), Acss3, Fasn and Scd1 were the highest in CONV (Figure 2C–E). On the other hand, the expression of the propionate transport protein Slc22a7 was not affected by microbial status. 

### 3.2. Cecal SCFA Pattern and Colonic Gene Expression Profile of Mice Harboring Different Microbial Status

#### 3.2.1. CHOW Fed Mice

**Figure 3:**
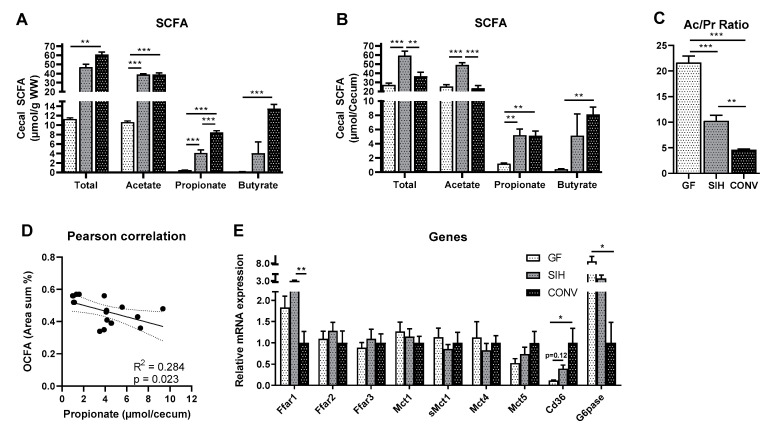
Conventional mice (CONV) on the CHOW diet have higher cecal propionate concentrations than germ-free mice (GF) and SIHUMI mice (SIH). Twenty-week-old male C3H/HeOuJ mice on CHOW diet (Altromin fortified 1314) harboring a germ-free (GF), simplified human (SIH) or conventional (CONV) microbiota: (**A**,**B**) short-chain fatty acid (SCFA) concentration in cecum and (**C**) the respective acetate/propionate ration (Ac/Pr). (**D**) Pearson correlation of cecal propionate concentration with hepatic odd-chain fatty acids (OCFA; C15:0 + C17:0) levels is shown. (**E**) mRNA expression in colon mucosa of genes involved in SCFA signaling, transport and intestinal gluconeogenesis. Data are mean + SEM (*n* = 4–6) and expression data were normalized to the CONV group. Rpl13a was used as the housekeeping gene. * *p* < 0.05; ** *p* < 0.01; *** *p* < 0.001.

Intestinal fermentation products, such as SCFA, have several effects on colonic function, for example, providing substrates for colonocytes or intestinal signaling. Here, we confirm that a conventional intestinal microbiota generates high concentrations of SCFA, but the ratio of SCFA was different according to colonization. As expected, the total cecal SCFA concentrations of mice on CHOW were the highest in conventional animals, with about 61 µmol/g WW, and the lowest in GF mice (11 µmol/g WW). Cecal acetate concentrations were not different between SIH and CONV mice, but were about 70% lower in GF mice. In contrast, the cecal propionate and butyrate concentrations increased with increasing microbiota diversity (Figure 3A). As the colonization status has a significant effect on total cecum weight (as shown in Table 1 and Table 2), we additionally calculated the total cecal amount of SCFA. This resulted in a different pattern compared to the calculation per gram wet weight. In terms of total cecum, the highest total SCFA and acetate amounts were detected in SIH mice, while levels were lower both in GF and CONV. Total cecal propionate and butyrate levels were not different between SIH and CONV, but they were significantly lower in GF (Figure 3B). Marked differences were obvious in the molar ratios (Figure 3C) of the two short-chain fatty acids acetate and propionate. The acetate/propionate ratio was highest in GF (22/1), and decreased with colonization complexity to 10/1 in SIH and to 5/1 in CONV. The total cecal propionate concentration was negatively correlated with hepatic OCFA level (Figure 3D).

Intestinal bacteria and their fermentation metabolites play an important role in the control of gene expression in the gastrointestinal tract, affecting the expressions of the genes that, among others, are involved in colonic signaling, SCFA transport and intestinal gluconeogenesis. Free fatty acid receptors (FFars), in particular Ffar1, Ffar2 and Ffar3, are expressed in enteroendocrine cells and are responsible for fatty acid (FA) sensing of the gastrointestinal tract. Ffar1, a medium- and long-chain FA-sensing G-protein-coupled receptor (GPCR), showed the highest gene expression in the colon mucosa of SIH mice, while the gene expressions of the SCFA receptors Ffar 2 and 3 were similar in all groups. The mRNA expression of monocarboxylate transporters (Mct 1, 4, 5) was determined in order to evaluate the intestinal transport of SCFA, which showed no differences between groups (Figure 3**E**). Further, the gene expression of Cd36 in colon mucosa was significantly increased in CONV compared to GF. In general, the intestinal mucosa is an important compartment for gluconeogenesis [21]. The gene expression of glucose 6-phosphatase (G6pase), a key enzyme of this pathway, showed a 10-fold increase in GF compared to CONV (Figure 3E). 

#### 3.2.2. HFI Fed Mice

**Figure 4:**
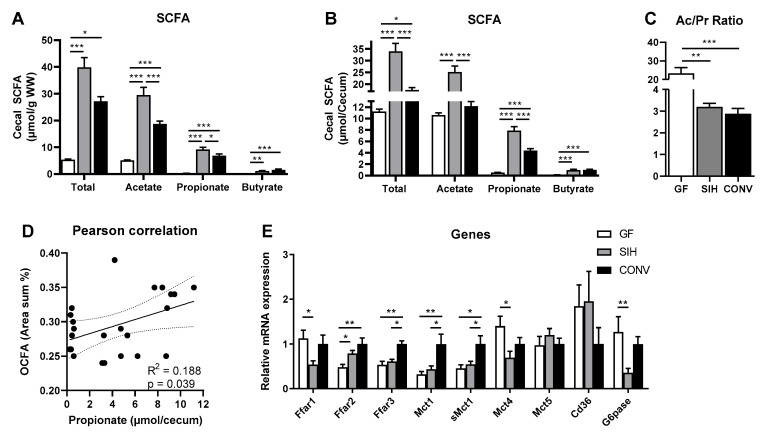
SIHUMI mice (SIH) on a high-fat inulin diet (HFI) display greater cecal acetate and propionate than conventional mice (CONV) and germ-free mice (GF). Sixteen-week-old male C3H/HeOuJ mice on a high-fat inulin diet (HFI, high-fat + 7% inulin): (**A**,**B**) short-chain fatty acid (SCFA) concentration in cecum and (**C**) the respective acetate/propionate ratio (Ac/Pr). (**D**) Pearson correlation of cecal propionate concentration with hepatic odd-chain fatty acids (OCFA; C15:0 + C17:0) levels is shown. (**E**) mRNA expression of colon mucosa of genes involved in SCFA signaling, transport and intestinal gluconeogenesis. Data are mean + SEM (*n* = 6–10) and expression data were normalized to the CONV group. Rpl13a was used as the housekeeping gene. * *p* < 0.05; ** *p* < 0.01; *** *p* < 0.001.

In contrast to the CHOW diet, supplementation with inulin (HFI) resulted in distinct cecal SCFA patterns that were similar in terms of both wet weight (Figure 4A) and total cecal content (Figure 4B). Of note, total cecal SCFA levels were considerably lower in HFI- than CHOW-fed mice, indicating the higher fermentability of the CHOW diet. Mice with SIHUMI colonization (SIH) on HFI displayed a higher total cecal SCFA content compared to GF and CONV, due to higher concentrations of acetate and propionate. Only butyrate concentrations were similar in the SIH and CONV groups, and these levels were higher compared to those in GF, which only showed minimal butyrate levels. Similar to CHOW feeding, however, the Ac/Pr ratio was highest in GF with 21/1, while it decreased to even lower levels (around 3/1) in SIH and CONV (Figure 4C). In this experiment, the cecal propionate amount was positively correlated with the OCFA content in the liver (Figure 4D), contrary to the CHOW-fed mice. Interestingly, feeding with an HFI diet led to more drastic colonization-dependent changes in mucosal gene expression than when feeding with the CHOW diet. The gene expression of all Ffars in the colon mucosa of HFI-fed mice was different between the groups. While the gene expression of the long-chain fatty acid receptor, Ffar1, was significantly lower in SIH compared to GF, the expressions of the short-chain fatty acid receptors Ffar2 and Ffar3 were highest in CONV and lowest in GF (Figure 4E). Mct1 and sMct1 are monocarboxylate transporters expressed in the apical membrane of the colonic epithelium. Both transporters showed an increased mRNA expression in CONV, compared to the GF and SIH groups, whereas the transporter in the basolateral membrane, Mct4, showed the highest expression in GF (Figure 4E). The G6pase gene expression was similar in GF and CONV, and was reduced in SIH (Figure 4E), which is supportive of a decreased intestinal gluconeogenesis in SIH. 

### 3.3. Fatty Acid Profile and Principle Component Analysis of Diets

**Figure 5:**
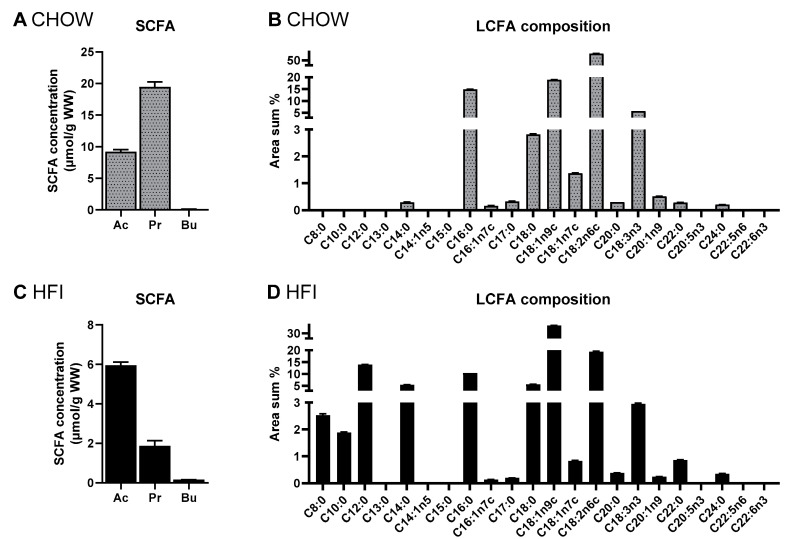
Fatty acid composition of diets used in these studies, measured by gas chromatography. Short-chain fatty acid (SCFA) concentration in (**A**) CHOW (Altromin fortified 1314) or (**C**) high-fat inulin diet (HFI, high-fat + 7% inulin). Long-chain fatty acid (LCFA) composition of the phospholipid fraction in the (**B**) CHOW or (**D**) HFI diet. Results are expressed as area percentage of individual fatty acids to total area of detected fatty acids. Data are mean + SEM (*n* = 3). Statistics were not assessed due to small sample size.

Assuming that there is no endogenous production of SCFA in mammals, the cecal amounts detected in GF should originate from the diet. The total cecal SCFA levels in GF were over twice as high on CHOW (25 µmol) than on the HFI diet (11 µmol). Therefore, we analyzed the long-chain fatty acid compositions and SCFA contents of the diets. The total SCFA content in the CHOW diet was over three times higher than in the HFI diet, and the former showed a different SCFA pattern. Importantly, the concentration of propionate was almost 10 times higher in the CHOW than in the HFI diet (Figure 5A,C). This is reflected in the cecal propionate content, which was over twice as high in GF on CHOW (1.19 µmol) than on HFI (0.51 µmol). Although differing in fat content, the LCFA composition of the two diets was quite similar. In both diets, no C15:0 and very low levels of C17:0 were detectable. However, the compositions of medium-chain fatty acids (C8:0, C10:0, C12:0, C14:0) were different. They were more abundant in the HFI diet, and almost non-detectable in CHOW (Figure 5B,D).

**Figure 6:**
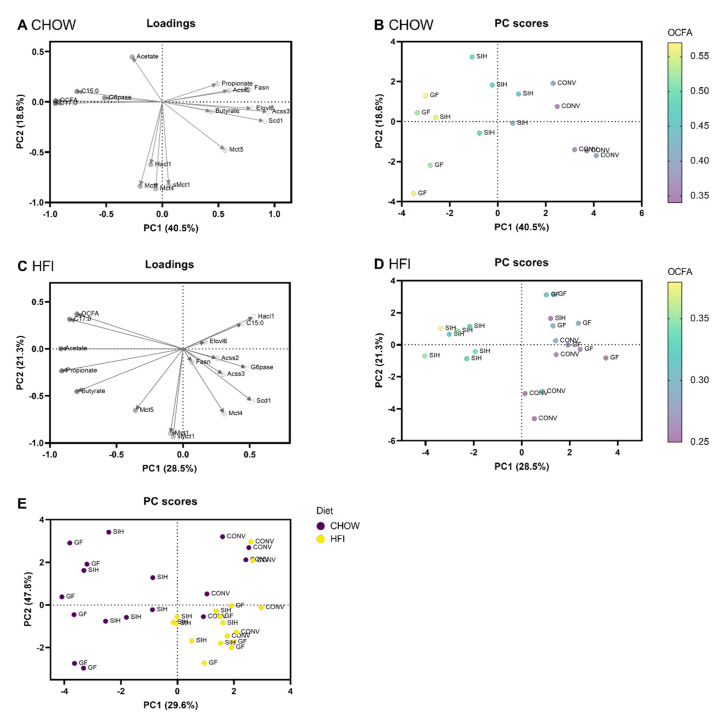
Principal component analysis (PCA) showing the loading plots and score plots of data, including OCFA formation, cecal SCFA concentrations and mRNA expression in the liver and intestines of male C3H/HeOuJ mice on (**A**,**B**) CHOW (Altromin fortified 1314) or (**C**,**D**) high-fat inulin diets (HFI, high-fat + 7% inulin) harboring a germ-free (GF), simplified human (SIH) or conventional (CONV) microbiota. (**E**) PC scores showing the distinct profile of CHOW-fed mice compared to HFI mice, independent of colonization status (GF vs. SIH vs. CONV).

In addition, a principal component analysis (PCA) was used to predict most diet-related or microbial-related changes in OCFA formation and hepatic lipid metabolism (Figure 6A–E). Specific patterns of correlation and clustering between the variables were visualized by loading plots. The loading plot of CHOW-fed mice revealed that OCFA formation (C15:0, C17:0, OCFA) and colonic G6pase mRNA expression were clustered together, indicating that they are positively correlated (Figure 6A). In comparison, the OCFA levels were negatively correlated with the cluster of lipogenic-related genes (Acss3, Elovl6, Scd1), as indicated by an angle of almost 180°. The score plot of CHOW mice indicated that the OCFA levels were dissimilar between groups, as reflected by the separation of the data into three clusters according to colonization status (Figure 6B).

In contrast, the loading plot of the HFI mice shows that hepatic C17:0 formation and cecal SCFA concentrations (acetate, propionate, butyrate) were strongly negatively correlated with PC1, whereas C15:0 clustered with hepatic Hacl1 gene expression and was positively correlated with PC1 (Figure 6C). The PC score plot of HFI-fed mice indicated that the OCFA levels of SIH were distinct from the other two groups (Figure 6D). To determine whether dietary or microbial effects were stronger, a combined score plot of data from the CHOW (dark purple) and HFI (yellow) mice was performed, which showed that the differences were mainly related to diet (CHOW vs. HFI) and marginally to microbial composition (GF vs. SIH vs. CONV) (Figure 6E). 

## 4. Discussion

Circulating odd chain fatty acids (OCFA) represent a minority of total fatty acid plasma concentrations in humans, but their higher presence is associated with a lower risk of numerous diseases, such as metabolic syndrome [22], type-2-diabetes [4,5,23] and nonalcoholic steatohepatitis [24]. Since it is still unclear whether increased OCFA are a cause or consequence of these health benefits, it is important to know which factors are contributing to controlling the levels of circulating OCFA. It is generally assumed that a dietary intake of milk fat increases circulating OCFA levels in humans because of their high production in ruminants [25,26]. Therefore, OCFA have long been used as a biomarker for dairy fat consumption. However, a valid biomarker is not, or is only slightly, affected by other nutrients, which is not the case with circulating OCFA. We and others have already shown that the intake of fermentable dietary fibers [12,27], the branched-chain amino acid valine [15], or fish oil [28] has an influence on the OCFA levels in the liver and in circulation. However, the importance of microbial-induced OCFA in rodents and humans is poorly understood, although the intestinal fermentation pattern and endogenous synthesis in the liver have obvious implications for inducing OCFA production. We have shown previously that gut-derived propionate, as both a supplement and a fermentation product, serves as a substrate for the de novo synthesis of OCFA in the liver, leading to elevated plasma OCFA concentrations in mice and humans [2,12]. This suggests the potential role of microbial species in affecting the formation of OCFA through their intestinal fermentation pattern, resulting in different amounts of SCFA, which can subsequently be metabolized in the large intestine and liver.

### 4.1. Effects of the Colonization Status on Hepatic Lipogenesis Are Not Dependent on the Diet

An effective method for differentiating host microbiome interactions is to compare animal models with a germ-free (GF), gnotobiotic SIHUMI or conventional (CONV) colonization, with varying degrees of complexity of the microbial composition, which thus induce varying capacities for intestinal fermentation. We employed a gnotobiotic mouse model, wherein mice were bred under germ-free conditions and colonized with simplified human microbial communities (SIH), to define specific in vivo effects. By placing these mouse models on different diets, we were able to show that GF and SIH mice show lower expression levels of the proteins involved in hepatic fatty acid uptake and lipogenic enzymes, which is a diet-independent effect. Both a CHOW diet and a semi-synthetic, high-fat inulin diet showed these effects, which were most pronounced in germ-free mice. The significantly altered key enzymes of lipid metabolism, which were differentially expressed in both studies, include CD36, ACSS3, FASN and SCD1, and these translocate fatty acids in the liver and catalyze the synthesis of saturated and monounsaturated fatty acids from acetyl-CoA and/or propionyl-CoA. It has been described that ACSS2 ligates acetate and CoA [29], whereas ACSS3 utilizes propionate to yield propionyl-CoA [30]. The ACSS3 gene and protein expression was consistently decreased in germ-free compared to conventional mice independently of the diet, suggesting a reduction in hepatic propionyl-CoA production from propionate in the absence of microbiota. However, the analysis of hepatic LCFA composition showed that hepatic OCFA incorporation was not correlated with ACSS3 expression, as discussed further in the next section. In general, our data show that an absence or lower complexity of microbial composition attenuates the expression of hepatic lipogenesis enzymes, without affecting total hepatic triglyceride concentrations. This is in line with previous studies on germ-free mice and antibiotic-treated animal models, which showed that the absence of microbes decreases hepatic fatty acid desaturation by SCD1 and elongation by ELOVL5 [31,32]. Further, in line with our data from CHOW-fed mice, it was shown that colonized animals have a higher FASN expression in the liver, which leads to increased hepatic triglyceride synthesis [33,34]. Germ-free mice consistently showed an increased cecal acetate/propionate ratio. Whether this might lead to increased acetyl-CoA production, driving an increase in fatty acid synthesis, is a matter of speculation.

### 4.2. Formation of Hepatic OCFA Is Affected by Microbial Composition and Fermentation Pattern, but Primarily by Dietary SCFA Content

Using mice with different levels of complexity in their microbial colonization and fed diets of substantially different compositions, we could show the highly complex interaction of diet components with microbiota as regards the hepatic lipid metabolism and the incorporation of OCFA. The dietary SCFA content also seems to have a significant impact on intestinal lipid and glucose metabolism.

In ruminants, it is known that their gut bacteria can synthesize OCFA via de novo synthesis by using propionyl-CoA instead of acetyl-CoA as the primer for fatty acid synthesis [35]. Since propionate is a C3 body, elongation through fatty acid synthase via the addition of malonyl-CoA (C2) results in the production of C15:0 and C17:0, instead of C16:0, as an end product. As such, it is possible that the intestinal production of propionate by gut bacteria is linked to the formation of OCFA in the liver, which has, to the best of our knowledge, not been considered previously. We addressed this by first determining the cecal SCFA levels of mice with different colonization statuses to induce differences in their production rates, assuming that the amount of SCFA is reflective of bacterial production. However, considering the observed differences in cecum weight, the total cecal SCFA levels were always the highest in SIH mice, while they showed no (CHOW) or only small (HFI) differences between GF and CONV. Total SCFA mainly reflects acetate levels, as the propionate and butyrate concentrations are much lower. In line with previous studies [36], the lowest cecal Ac/Pr ratios were detectable in mice with a complex bacterial composition due to their increased bacterial propionate production, which is of course absent in germ-free mice. Nevertheless, cecal propionate was detectable in GF at levels two times higher when on CHOW than in those on HFI. This can be explained by the high propionate content of the CHOW diet, which was around 10-fold higher than in the semi-synthetic HFI diet, while the acetate concentrations were rather similar. Higher hepatic OCFA levels in GF together with the inverse association of hepatic OCFA with cecal propionate levels in CHOW-fed mice suggest the different metabolic fates of dietary versus fermentation-derived propionate. 

In general, SCFAs play different roles in intestinal and systemic metabolism. Butyrate is preferably used as a major energy source for colonocytes, whereas acetate and propionate are important regulators of hormone production and are substrates for energy metabolism. More than 60% of propionate is apparently used for whole-body glucose production [37], as it can be converted into propionyl-CoA and succinyl-CoA, which enters the TCA cycle and results in the formation of pyruvate for gluconeogenesis [38]. It was shown that intestinal gluconeogenesis (IGN) has beneficial effects on glucose homeostasis, since the induction of IGN is associated with an increased glucose tolerance and insulin sensitivity. In mice deficient in IGN (by intestinal disruption of glucose-6-phosphatase, G6pase), these positive effects were abolished [39]. In the present study, the intestinal expression of G6pase was 10-fold higher in GF and 4.5-fold higher in SIH mice, compared to CONV on a CHOW diet. These data indicate a high conversion of propionate to glucose by IGN in germ-free and SIHUMI, which could improve their energy and glucose metabolism. Furthermore, the loading plot supports the idea of a high propionate turnover, as the intestinal G6pase expression and hepatic OCFA levels are clustered together, showing a high correlation. 

In contrast, fatty acid translocase (CD36), which is abundantly expressed along the digestive tract of mice and binds long-chain fatty acids [40], was upregulated in CONV on CHOW. The increased mucosal CD36 expression in the colon of CONV suggests that a complex microbiota promotes intestinal fatty acid uptake, possibly leading to increased adiposity, which is consistent with the highest body weight being seen in this group. These data are in line with those form a study comparing germ-free and conventional mice, which showed that a complex composition of gut bacteria in mice increased the intestinal absorption of lipids, resulting in marked obesity [41]. 

Overall, our data indicate that on a CHOW diet, cecal produced propionate is not preferentially used for fatty acid synthesis. Although the propionate concentration was lower in GF and SIH mice, it seems to be used as a substrate for intestinal gluconeogenesis and OCFA formation, whereas mice with a complex colonization displayed increased long-chain fatty acid uptake, which may induce TG accumulation and obesity in the long-term. Fiber fermentation, and thus SCFA production, in mice occurs mainly in the cecum, with the subsequent uptake of SCFA in the colon. Dietary SCFA can be absorbed from the small intestine into the portal vein. This could explain the high levels of hepatic OCFA formation in germ-free mice on CHOW, considering the high propionate levels in CHOW. High dietary concentrations of SCFA may override the effects of SCFA produced in the cecum. 

Feeding with a semi-synthetic high-fat (HFI) diet supplemented with the highly fermentable fiber inulin resulted in a very different association between cecal SCFA and hepatic OCFA compared to the CHOW diet. The fiber-rich HFI diet was used to induce changes in the microbial fermentation pattern, and in fact, cecal propionate as well as total hepatic OCFA were the highest in SIH mice, without any difference between GF and CONV. This is in line with other data showing that a germ-free and conventional colonization does not affect circulating OCFA levels if mice are on semi-synthetic low-fat or high-fat diets [16]. However, our gnotobiotic SIHUMI mouse model, in combination with an inulin-rich diet, was able to induce cecal propionate production in the cecum, as highlighted before [18], which here is also reflected in the increased levels of OCFA in the liver, as supported by their positive correlation and clustering in the PCA. Based on these data, we speculate that one species of SIHUMI had an increased capacity to produce propionate, which is not the case in conventional mice because of intraspecific concurrence or absence. From the loading plots, it is also evident that feeding with an HFI diet exclusively affected the formation of C17:0, whereas the C15:0 levels were lower and correlated with hepatic Hacl1 expression, which is a key enzyme related to a-oxidation of even-chain fatty acids. Here, the effect is predominant, because the SCFA concentration in the HFI diet is 5-fold lower than the concentration in the cecum, and thereby the latter is not masked by the high dietary SCFA input. Further, the gene expression of G6pase in the colon mucosa of inulin-fed SIH mice was reduced, suggesting that the produced propionate is not used for intestinal gluconeogenesis, but is rather incorporated into fatty acid synthesis. 

Taken together, our data provide evidence that hepatic OCFA formation is affected by both diet and microbial composition. If the amount of SCFA in the diet is already very high, the cecal production of propionate does not seem to affect OCFA levels. For mice on a semi-synthetic diet with low SCFA concentrations, cecal propionate production is correlated with hepatic OCFA levels, suggesting that endogenously produced propionate can be used for de novo lipogenesis. Whether certain bacteria of the SIHUMI consortium are responsible for the induction of cecal propionate and the resulting increase in OCFA should be clarified in further studies. This could be helpful in understanding how propionate and OCFA levels could also be endogenously increased in humans, possibly affecting the incidence of diseases, as described in epidemiological studies. Since our data provide evidence that hepatic OCFA formation is affected by diet and microbial composition, the use of OCFA as a marker of dairy fat intake should be reconsidered.

## Figures and Tables

**Table 1:** Phenotypical changes of male C3H/HeOuJ mice at an age of 20 weeks on CHOW diet with different colonization states. GF = germ-free mice, SIH = SIHUMI mice, CONV = conventional mice. Data are mean +/− SEM (*n* = 6). Different letters indicate significant differences between groups.

	GF	SIH	CONV	*p*-Value
**Phenotype**				
Final body weight (g)	31.3 ± 0.9 ^a^	37.4 ± 0.7 ^b^	37.5 ± 1.7 ^b^	<0.01
**Tissue weights**				
Liver (g)	1.23 ± 0.05 ^a^	1.61 ± 0.03 ^b^	2.13 ± 0.16 ^c^	<0.05
Quadriceps (g)	0.36 ± 0.02	0.39 ± 0.01	0.36 ± 0.03	ns
Gastrocnemius (g)	0.27 ± 0.01 ^a^	0.30 ± 0.01 ^a,b^	0.34 ± 0.02 ^b^	<0.05
Cecum (g)	2.39 ± 0.14 ^a^	1.23 ± 0.08 ^b^	0.60 ± 0.05 ^c^	<0.001
sWAT (g)	0.27 ± 0.04 ^a^	0.48 ± 0.03 ^a,b^	0.73 ± 0.12 ^b^	<0.01
eWAT (g)	0.27 ± 0.03 ^a^	1.08 ± 0.09 ^b^	1.21 ± 0.20 ^b^	<0.01
BAT (g)	0.05 ± 0.00 ^a^	0.07 ± 0.01 ^a^	0.14 ± 0.02 ^b^	<0.05

**Table 2:** Phenotypical changes in male 16-week-old C3H/HeOuJ mice with different colonization states fed a high-fat + inulin diet (HFI) for 4 weeks. GF = germ-free mice, SIH = SIHUMI mice, CONV = conventional mice. Data are mean +/− SEM (*n* = 10). Different letters indicate significant differences between groups.

	GF	SIH	CONV	*p*-Value
**Phenotype**				
Final body weight (g)	29.8 ± 0.6	30.6 ± 0.5	30.8 ± 0.5	ns
Final fat mass (g)	2.99 ± 0.32	3.90 ± 0.35	3.16 ± 0.41	ns
Final lean mass (g)	26.8 ± 0.3 ^a,b^	26.7 ± 0.2 ^a^	27.7 ± 0.3 ^b^	<0.05
Food intake (g/d)	3.63 ± 0.11	3.72 ± 0.12	3.72 ± 0.11	ns
**Tissue weights**				
Liver (g)	1.41 ± 0.06 ^a^	1.45 ± 0.04 ^a^	1.75 ± 0.03 ^b^	<0.001
Quadriceps (g)	0.36 ± 0.01	0.34 ± 0.01	0.35 ± 0.01	ns
Gastrocnemius (g)	0.27 ± 0.01 ^a,b^	0.25 ± 0.01 ^a^	0.29 ± 0.01 ^b^	<0.05
Cecum (g)	2.10 ± 0.07 ^a^	0.85 ± 0.04 ^b^	0.66 ± 0.03 ^c^	<0.05
sWAT (g)	0.23 ± 0.03 ^a^	0.40 ± 0.03 ^b^	0.37 ± 0.03 ^b^	<0.05
eWAT (g)	0.31 ± 0.06 ^a^	0.70 ± 0.06 ^b^	0.50 ± 0.07 ^a,b^	<0.001
BAT (g)	0.07 ± 0.01 ^a^	0.07 ± 0.01 ^a^	0.12 ± 0.01 ^b^	<0.001

## Data Availability

The data presented in these studies are available on request from the corresponding author.

## Supplementary Materials

The following are available online at https://www.mdpi.com/article/10.3390/nu13051546/s1, Table S1: Oligonucleotides used in these studies to measure mRNA levels.
