# Supplementary material for: Effect of Microbial Status on Hepatic Odd-Chain Fatty Acids Is Diet-Dependent
Source: Nutrients. 2021 May 4;13(5):1546. doi: 10.3390/nu13051546 (PMC8147859; doi:10.3390/nu13051546)
Supplement: Supplementary file 1 [file nutrients-13-01546-s001.zip › nutrients-1201662-supplementary.pdf]

Supplementary Files

Table S1. Oligonucleotides used in these studies to measure mRNA levels.

Primer name		Primer sequence (5′→3′)
Hprt	f	GTT GAA GAT ATA ATT GAC ACT GGT AAA ACA
	r	AGC TTG CAA CCT TAA CCA TTT TG
Actin	f	GCC AAC CGT GAA AAG AGA C
	r	TAC GAC CAG AGG CAT ACA G
Rpl13a	f	AGC AGA TCT TGA GGT TAC GGA
	r	TTA TTG GGT TCA CAC CAG GA
Cd36	f	CCA AGC TAT TGC GAC ATG AT
	r	ACA GCG TAG ATA GAC CTG CAA A
Acss2	f	TGT GTG ATG GGC CAT ACC TTC
	r	GTA GTC TGG TGT GGC AAT GG
Acss3	f	ACC AGG AAG GAA GGT GGA GT
	r	AAC TCT GTC TGG CCT GTG CT
Fasn	f	TTG ATG ATT CAG GGA GTG GA
	r	TTA CAC CTT GCT CCT TGC TG
Elovl6	f	TGC AGG AAA ACT GGA AGA AGT CT
	r	AGC GGC TTC CGA AGT TCA A
Scd1	f	TTC TTC TCT CAC GTG GGT TG
	r	CGG GCT TGT AGT ACC TCC TC
Ppara	f	ATT CGG CTG AAG CTG GTG TA
	r	AAG CGA ATT GCA TTG TGT GA
Hacl1	f	ACA GGC TTG ATG CAG GTT CT
	r	CCA CTT CCA TGC CAG AAA AT
Ffar1	f	GGC CCT ATA ATG CCT CCA AT
	r	CCC TGT GAT GAG TCC CAA CT
Ffar2	f	CTT CCC GGT GCA GTA CAA GT
	r	GCT CTT GGG TGA AGT TCT CG
Ffar3	f	TCC TCA GCA CCC TCA ACT CT
	r	CTA GCT CGG ACA CTC CTT GG
Mct1	f	TGT TAG TCG GAG CCT TCA TTT C

	r	CAC TGG TCG TTG CAC TGA ATA
sMct1	f	CCA CTT CTG GGC TTG TTT TC
	r	CAG ACC AGT GAG TGC TCC AA
Mct4	f	CTG AGG CAC GAG CAA GAG TA
	r	GGC TGC TTT CAC CAA GAA CTG
Mct5	f	CCA TTA TGT CAT CAC TCC GTT TC
	r	CTC TCC AAG TAC GTC ACA GAT GA
G6pase	f	ACG TAT GGA TTC CGG TGT TT
	r	GGT AGA TCC GGG ACA GAC AG
